# Influence of inorganic nanoparticles on dental materials’ mechanical properties. A narrative review

**DOI:** 10.1186/s12903-023-03652-1

**Published:** 2023-11-21

**Authors:** Ghada Naguib, Abdulrahman A. Maghrabi, Abdulghani I. Mira, Hisham A. Mously, Maher Hajjaj, Mohamed T. Hamed

**Affiliations:** 1 Department of Restorative Dentistry, Faculty of Dentistry, King Abdulaziz University, Jeddah, Saudi Arabia; 2 Department of Oral Biology, Cairo University School of Dentistry, Cairo, Egypt; 3 King Abdulaziz University Dental Hospital, Jeddah, Saudi Arabia; 4 Department of Oral and Maxillofacial Prosthodontics, Faculty of Dentistry, King Abdulaziz University, Jeddah, Saudi Arabia; 5 Department of Fixed Prosthodontics, Cairo University School of Dentistry, Cairo, Egypt

**Keywords:** Inorganic nanoparticles, Mechanical properties, Dental materials, Nanotechnology, Dentistry

## Abstract

Inorganic nanoparticles have been widely incorporated in conventional dental materials to help in improving their properties. The literature has shown that incorporating nanoparticles in dental materials in different specialties could have a positive effect on reinforcing the mechanical properties of those materials; however, there was no consensus on the effectiveness of using nanoparticles in enhancing the mechanical properties of dental materials, due to the variety of the properties of nanoparticles itself and their effect on the mechanical properties. This article attempted to analytically review all the studies that assessed the effect of different types of inorganic nanoparticles on the most commonly used dental materials in dental specialties such as polymethyl methacrylate, glass ionomer cement, resin composite, resin adhesive, orthodontic adhesive, and endodontic sealer. The results had shown that those inorganic nanoparticles demonstrated positive potential in improving those mechanical properties in most of the dental materials studied. That potential was attributed to the ultra-small sizes and unique physical and chemical qualities that those inorganic nanoparticles possess, together with the significant surface area to volume ratio. It was concluded from this comprehensive analysis that while a definitive recommendation cannot be provided due to the variety of nanoparticle types, shapes, and incorporated dental material, the consensus suggests using nanoparticles in low concentrations less than 1% by weight along with a silane coupling agent to minimize agglomeration issues and benefit from their properties.

## Introduction

Human tooth tissues are comprised of diversified micro nanostructures. For example, carbonated hydroxyapatite, with a range of 10–200 nm in size, is made up of 96% of enamel structure [1]. Dentin nanostructures are also unique, with intertubular dentin being 60 nm long and 2–5 nm thick. Peritubular dentin nanostructures are roughly 25 nm long and 2–5 nm thick, and dentine collagen fibrils range in size from 20 to 75 nm [1, 2].

Nanotechnology is essentially a science and engineering of functional systems at a nanoscale (one-billionth of a meter) [3–6]. Accordingly, when a material is smaller than 100 nm in one dimension, it is defined as a nanomaterial [3–5, 7, 8]. Hence, the aim of nanotechnology in dentistry is to mimic the natural tissue architecture, both soft and hard, by adapting new dental biomaterials to achieve better restoration of lost tissue that occurs due to disease, and to provide antimicrobial activity where necessary [1].

Nanomaterials can be synthesized in a variety of ways depending on a multitude of factors, such as the dimension of the materials being created. Hu and Shaw categorized nanoparticles as zero-dimensional, one-dimensional, two-dimensional, and three-dimensional [9]. Zero-dimensional nanoparticles (NPs) are defined as the nanostructure that has all dimensions in the nano-range, and they are amongst the most commonly employed type of nanomaterials in dentistry [9].

The main characteristic of NPs is that they have a very potent antimicrobial action against bacterial biofilm [10]. As NPs have a high surface area and high charge density, the nanoparticle ions in contact with microorganisms produce a germicidal effect [10–13]. They can also fill the gaps between the inter-polymeric chains, resulting in augmented mechanical and physical strength [14].

Different types of NPs have different properties. There are two main types of NPs: organic and inorganic. Quaternary ammonium polyethyleneimine (QPEI) [15], quaternary ammonium dimethacrylate [16], dimethylaminohexadecyl methacrylate [17] and chitosan [18, 19] are examples of organic NPs. Metal and metal oxides are examples of inorganic NPs.

Meanwhile, polyhedral oligomeric silsesquioxane (POSS) is an example of a nanosized organic-inorganic hybrid material consisting of a Si-O bond in three-dimensional architecture that is used with dental nanocomposites [20, 21].

Studies have shown that both organic and inorganic NPs have antimicrobial properties. The mechanical and physical properties have been more extensively studied with inorganic NPs, whereas there is limited data on the mechanical and physical properties of organic NPs. Similar to NPs, the large surface to volume ratio of nanomaterials is one of their most notable characteristics [7]. When considering a nanoparticle with a diameter of a few nm, all the atoms are either at the nanoparticle’s surface or inside the particle within a few atomic distances from the surface, depending on the atoms’ size and the nanoparticle’s size [7]. All atoms in a bulk material bind to their neighbors. Surface atoms, on the other hand, have fewer near neighbors, resulting in hanging or unsatisfied bonds, which results in an extra energy called surface energy, surface free energy, or surface tension that has a significant impact on the particle’s physical properties [16]. The above-mentioned physical and chemical phenomena have a number of significant implications for the characteristics of nanoscale materials and their manufacturing. In order to lower their surface energy, NPs try to agglomerate, resulting in the loss of its optical properties [4, 5, 7, 22]. It also loses some of its effective characteristics like the antimicrobial action, or it can leave inter-polymeric chain spaces unfilled, reducing the unique mechanical features. For this reason, good dispersion of NPs in the matrix is considered the main key for an effective nanomaterial. Similarly, the biological safety of nanoparticles is a critical aspect, as it depends on several factors such as their shape, size, method of preparation, and the chemicals used [23].

Recently, NPs are purposefully embedded in dental products to improve the material’s qualities as well as improve its longevity and success rate [3, 24]. Such fillers can be found in a variety of dental materials, including resin-based composites, cements, and impression materials [8, 12, 13] for the treatment of common dental diseases such as caries and periodontal infections [1, 25, 26]. As patient treatment needs, both esthetic and function, continue to expand, today’s scientists and engineers need to diversify nanoscale materials to take advantage of their superior features [1, 27].

Many studies and reviews focus on the antimicrobial properties of NPs-reinforced materials and demonstrated that NPs possess superior antimicrobial activity compared to their regular sized counterparts [10–13, 16, 28–41]. On the other hand, the studies that evaluate the physical properties of NPs are limited and the results presented are conflicted [42–44]. Inorganic nanoparticles incorporated into dental materials have been evaluated for their potential benefits, but the body of literature that focuses on the mechanical properties and their importance specifically to dental materials is lacking. In response to this, we sought to provide a comprehensive analytical review on how inorganic NPs influence the mechanical properties of dental materials in a variety of dental applications including prosthodontics, orthodontics, restorative dentistry, and endodontics.

## Methodology

In order to achieve a conclusive result regarding the effectiveness of NPs on the mechanical properties of different dental materials, three databases (PubMed, Web of Science and Scopus) were searched during the time period from January 2021 to December 2021 using the association of the keywords: ‘inorganic nanoparticles,’ ‘dentistry’ and ‘mechanical properties’. The Boolean operators for each database were written as [“inorganic nanoparticles” AND “dentistry” AND “mechanical properties”]. For an article to be considered for inclusion in the review, it had to be written in English, and performed as a laboratory study or on humans (in vivo or in vitro on human cells). Furthermore, the papers had to be done on materials that were reinforced by NPs, and the test must have involved the evaluation of the mechanical properties of these NPs-reinforced materials.

**Fig. 1:**
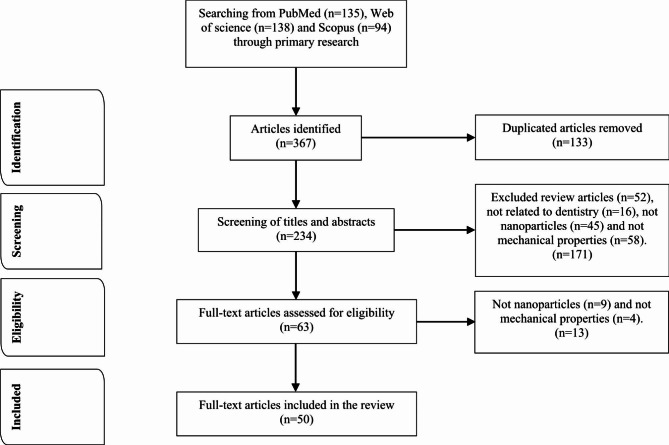
Visual diagram of article filtration and selection from the PubMed, Web of Science, and Scopus databases

The initial search in PubMed, Web of Science and Scopus databases conducted by the keywords revealed a total of 367 articles (Fig. 1). The screened data resulted in 133 duplicated articles. 234 articles remained to be subjected to title and abstract analysis. The eligibility criteria involved original articles in the English language related to NPs incorporated with conventional dental materials and the measurement of the mechanical properties. Review articles were excluded. This resulted in 63 articles of which 13 were excluded after reading the full text. Finally, full texts of 50 articles were included in the review.

## Results

The total number of full-text articles included in this review is 50 articles. All tables show results of different parameters including author and year of the publication, sample size, NPs used in the dental material, size, concentration, the type of dental material, treatment groups, type of mechanical test, and outcome/result findings. Tables 1, 2, 3, 4 and 5, and 6 show results for the review of the materials: polymethyl methacrylate, glass ionomer cement, resin composite, resin adhesive, orthodontic adhesive, and endodontic sealer, respectively.

**Table 1:** Mechanical properties of PMMA reinforced by different NPs.

Author (Year)	Sample(s)	Type of NPs And Materials Used	Treatment Group(s)	Type of Tests	Outcomes
da Silva et al. (2012) [45]	*N* = 50 specimens. 5 groups.	• Silicon dioxide SiO_2_. • Microwave-polymerized PMMA.	• One control group. • Four groups contain PMMA with silane surface treated SiO_2_ at 0.1, 0.5, 1 and 5 wt%, respectively.	• Flexural strength. • Hardness.	• Addition of 0.5 and 1 wt% SiO_2_ resulted in significantly higher flexural strength than control group. • Addition of 5 wt% SiO_2_ resulted in decreased flexural strength. • Control group showed the highest mean hardness value. The addition of SiO_2_ from 0.1 to 5 wt% led to gradually decreasing hardness values.
Balos et al. (2014) [46].	*N* = 105 specimens. 21 groups.	• Silicon dioxide SiO_2_. Size of 7 nm. • Three commercially types of heat-polymerized PMMA.	• One control group for each material. • Six groups contain PMMA with SiO_2_ at 0.023%, 0.046%, 0.092%, 0.23%, 0.46% and 0.92%, respectively for each material.	• Fracture toughness. • Microhardness.	• When compared to the control group, all experimental groups had higher fracture toughness and microhardness. • The maximum value was observed for SiO_2_ concentrations of 0.023%. The fracture toughness and microhardness readings rapidly declined as the SiO_2_ content increased.
Cevik et al. (2016) [47].	*N* = 40 specimens. 5 groups.	• Silicon dioxide SiO_2_. • Prepolymer NPs. • Heat-polymerized PMMA.	• One control group. • Two groups contain PMMA with SiO_2_ at 1 and 5 wt%, respectively. • Two groups contain PMMA with polymer at 1 and 5 wt%, respectively.	• Flexural strength. • Microhardness.	• The control group had significantly better flexural strength than the SiO_2_ and polymer groups. • Adding 5 wt% SiO_2_ to PMMA enhanced its hardness but it was not statistically significant.
Rashahmadi et al. (2017) [48].	*N* = 9 specimens. 9 groups.	• Silicon dioxide SiO_2_. Size of 20–30 nm. • Titanium dioxide TiO_2_. Size of 20 nm. • Aluminium oxide Al_2_O_3_. Size of 20 nm. • PMMA.	• One control group. • Three groups contain PMMA with TiO_2_ at 0.5, 1 and 2 wt% respectively. • Three groups contain PMMA with SiO_2_ at 0.5, 1 and 2 wt% respectively. • Two groups contain PMMA with Al_2_O_3_ at 0.5 and 1 wt% respectively.	• Flexural strength. • Impact strength. • Young’s modulus. • Hardness.	• Addition of 0.5 wt% TiO_2_ increased flexural strength. With the addition of 1 and 2 wt% TiO_2_, it is nearly identical to pure PMMA. The addition of SiO_2_ and Al_2_O_3_ significantly reduced it. • Adding 0.5, 1, and 2 wt% TiO_2_ enhanced the impact strength. Impact strength was unaffected by SiO_2_ and Al_2_O_3_ in concentrations of 0.5 and 1 wt%. However, it was higher in the group with 2 wt% SiO_2_. • The results showed that samples with 2 wt% TiO_2_ and SiO_2_ improved their Young’s modulus and hardness.
Topouzi et al. (2017) [49].	*N* = 81 specimens. 9 groups	• Silicon dioxide SiO_2_. Size of 12 nm. • Trietoxyvinylsilane. • Auto-polymerized PMMA.	• One control group. • Three groups contain PMMA with silanized SiO_2_ (T-SIL) at 0.25, 0.050, 0.75 and 1 wt% respectively. • Three groups contain PMMA with unsilanized SiO_2_ (SIL) at 0.25, 0.050, 0.75 and 1 wt% respectively.	• Fracture toughness.	• In comparison to the control group, adding 0.25, 0.50, 0.75 and 1 wt% to SIL and T-SIL increased the fracture toughness significantly. • The PMMA reinforced with 0.25 wt% T-SIL had the highest fracture toughness.
Karci et al. (2019) [49].	*N* = 210 specimens. 30 groups.	• Silicon dioxide SiO_2_. Size of 15 nm. • Aluminium oxide Al_2_O_3_. Size of 18 nm. • Titanium dioxide TiO_2_. Size of 13 nm. • Heat-polymerized PMMA. • Auto-polymerized PMMA. • Microwave PMMA.	• One control group for each material. • Three groups contain PMMA with SiO_2_ at 1, 3 and 5 wt%, respectively for each material. • Three groups contain PMMA with TiO_2_ at 1, 3 and 5 wt%, respectively for each material. • Three groups contain PMMA with Al_2_O_3_ at 1, 3 and 5 wt%, respectively for each material.	• Flexure strength.	• The heat-polymerized group exceeded the auto-polymerized and microwave groups in terms of flexural strength. • The flexural strength values of groups with 1 wt% statistically significantly exceed those of the control group in heat and auto-polymerized groups. • There were no statistically significant differences between the microwaved groups.
Gad et al. (2020) [50].	*N* = 260 specimens. 13 groups.	• Silicon dioxide SiO_2_. Size of 12 nm. • Zirconium dioxide ZrO_2_. Size of 14 nm. • Diamond DNPs. Size of 19 nm. • Heat-polymerized PMMA.	• One control group. • Four groups contain PMMA with SiO_2_ at 0.5, 1, 2.5 and 5 wt% respectively. • Four groups contain PMMA with ZrO_2_ at 0.5, 1, 2.5 and 5 wt% respectively. • Four groups contain PMMA with DNPs at 0.5, 1, 2.5 and 5 wt% respectively.	• Surface hardness.	• There was a significant increase in hardness between all reinforced groups throughout all experimental groups, and this increase was concentration dependent. • When compared to the ZrO_2_ and DNPs groups, SiO_2_ had the highest hardness values per concentration.
Zhang et al. (2014) [51].	*N* = 102 specimens. 33 groups.	• Zirconium oxide ZrO_2_. Size of 90 nm. • Aluminium borate whiskers (ABW). • PMMA.	• Pure PMMA as blank group. • Four experimental groups contain PMMA with silanized ZrO_2_ at 1, 2, 3, and 4 wt%, respectively. Each group was subdivided according to ZrO_2_/ABW mass ratios of 2:1, 1:1, 1:2, and 1:3. • Four experimental groups contain PMMA with unsilanized ZrO_2_ at 1, 2, 3, and 4 wt%, respectively. Each group was subdivided according to ZrO_2_/ABW mass ratios of 2:1, 1:1, 1:2, and 1:3.	• Flexure strength. • Microhardness.	• Flexural strength was significantly increased by ZrO_2_-ABW at 1 and 2 wt% only. • Silanized ZrO_2_-ABW showed higher flexural strength when compared with the unsilanized. • Regarding ZrO_2_/ABW ratio, with an increase in the ZrO_2_/ABW ratio, the flexural strength increased at first, then decreased.
Gad et al. (2016) [52].	*N* = 180 specimens. 9 groups.	• Zirconium oxide ZrO_2_. Size of 90 nm. • Heat-polymerized PMMA. • Auto-polymerized PMMA.	• One control group of heat-polymerized. • Two repair groups divided according to the surface design of repair area into four butt joint plate groups and four bevel joint plate groups (one unreinforced auto-polymerized PMMA group and three groups contain auto-polymerized PMMA with ZrO_2_ at 2.5, 5, and 7.5 wt%, respectively).	• Flexure strength. • Impact strength.	• Except for the bevel group reinforced with 7.5 wt% ZrO_2_, the control group’s flexural strength was significantly higher than other repaired groups. • A significant difference between ZrO_2_ reinforced auto-polymerized groups and unreinforced group. • Regarding impact strength, the mean values of all repaired groups were significantly lower than those of the control group. • In comparison to the unreinforced group, the butt group with 2.5 wt% had a significant increase in impact strength, while the bevel group with 7.5 wt% had a significant drop.
Alhavaz et al. (2017) [53].	*N* = 80 specimens. 4 groups.	• Zirconium oxide ZrO_2_. Size of 15 nm. • Auto-polymerized PMMA.	• One control group. • Three groups contain PMMA with ZrO_2_ at 1, 2.5 and 5 wt%, respectively.	• Flexure strength. • Microhardness.	• Experimental groups at all concentrations show an increase in the flexure strength and microhardness. • The flexural strength significantly increased only in the group with 2.5 wt%. • The microhardness value is significantly increased in the groups with 2.5 and 5 wt%.
Ergun et al. (2018) [54].	*n* = 160 specimens. 4 groups.	• Zirconium oxide ZrO_2_. Size of 15 nm. APTES silane. Heat-polymerized PMMA.	• One control group. • Three groups contain PMMA with ZrO_2_ at 5, 10 and 20 wt%, respectively.	• Flexure strength. • Microhardness.	• Experimental groups at all concentrations show a significant decrease in the flexure strength. • The highest microhardness value showed in the group with 5 wt%.
Gad et al. (2018) [55].	*n* = 40 specimens. 4 groups.	• Zirconium oxide ZrO_2_. Size of 40 ± 2 nm. • Heat-polymerized PMMA.	• One control group. • Three groups contain PMMA with ZrO_2_ at 2.5, 5 and 7.5 wt%, respectively.	• Tensile strength.	• Experimental groups at all concentrations show a significant increase in the tensile strength when compared with the control group—the rise is concentration dependent.
Elmadani et al. (2019) [56].	*N* = 70 7 groups.	• Zirconium oxide ZrO_2_. Size of 100 nm. • MEMO Silane. • Polystyrene (PS). • PMMA.	• One control group. • One group contains 1 wt% ZrO_2_, one group contains 1 wt% ZrO_2_/MEMO, one group contains 2.5 wt% PS, one group contains 2.5 wt% PS with 1 wt% ZrO_2_, one group contains 2.5 wt% PS with 0.5 wt% ZrO_2_/MEMO and one group contains 2.5 wt% PS with 1 wt% ZrO_2_/MEMO.	• Microhardness. • Impact strength.	• The addition of 1 wt% ZrO_2_ improved microhardness by 3%, while using silane with ZrO_2_ led to increasing the hardness up to 29%. • The use of PS reduces the hardness of the material. • The PS group had the best ability to absorb energy during the impact—nearly 92% better than pure PMMA.
Sodagar et al. (2012) [57].	*N* = 90 specimens. 6 groups.	• Silver Ag. Size of 38 nm. • 2 types of auto-polymerized PMMA	• One control group for each material. • Two groups contain 0.05 and 0.2 wt% Ag, respectively for each material.	• Flexure strength.	• The addition of 0.05 wt% to Rapid Repair significantly reduced the flexural strength, while the addition of 0.2 wt% restored the strength to that of the control group. • There was no significant increase in flexural strength in Selecta Plus at both 0.05 and 0.2 wt%.
Koroglu et al. (2016_)_ [59].	*N* = 56 specimens. 8 groups.	• Silver Ag. Size of 68 nm. • Heat-polymerized PMMA. • Microwave-polymerized PMMA.	• One control group for each material. • Three groups contain 0.3, 0.8 and 1.6 wt% Ag, respectively for each material.	• Flexure strength. • Impact strength.	• For the heat-polymerized group the addition of Ag had no effect on flexure strength. • For the microwave-polymerized the addition of 0.3 wt% Ag increased the flexure strength and elastic modulus but not significantly. For the addition of 0.8 and 1.6 wt%, there was a significant reduction in the flexure strength and elastic modulus. • The addition of Ag had no effect on the impact strength for both group.
Munikamaiah et al. (2018) [58].	*N* = 60 specimens. 6 groups.	• Silver Ag. Size of 10–20 nm. • PMMA.	• Short and long curing cycles (one control group, two groups contain 0.5% and 5% by volume Ag, respectively).	• Flexure strength.	• When compared to the control group, 0.5% Ag had the highest and 5% Ag had the lowest mean flexural strength in both curing cycles.
Bacali et al. (2020) [59].	*N* = 30 specimens. 3 groups.	• Graphene silver G-Ag. • PMMA.	• One control group. • Two groups contain 1 and 2 wt% G-Ag, respectively.	• Flexure strength.	• Flexural strength was significantly higher in the G-Ag groups than in the control group, and the increase was concentration dependent.
Kumar et al. (2019) [60].	*N* = 80 specimens. 4 groups.	• Titanium dioxide TiO_2_. • Heat-polymerized PMMA. • Microwave-polymerized PMMA.	• Two control groups contain unreinforced PMMA processed using water bath (group I) and microwave (group III) techniques. • Two groups contain 1 wt% TiO_2_ processed using water bath (group II) and microwave (group IV) techniques.	• Impact strength.	• Impact strength of group IV was the highest and group I was the lowest. • Only group I had a significant difference in strength when compared to all other groups individually.
Protopapa et al. (2011) [61].	*N* = 121 specimens. 5 groups	• Diamond DNPs. Size of 20–60 nm. • Auto-polymerized PMMA.	• One control group. • Four groups contain 0.1, 0.38, 0.5 and 0.83 wt% DNPs, respectively.	• Fracture toughness. • Impact strength.	• Fracture toughness enhanced as the DNPs content increased up to 0.38 wt%, albeit the difference was only for 0.1 wt% as statistically significant when compared to the control group. • The control group had the lowest impact strength, while 0.1 wt% had the highest statistically significant value, followed by 0.83 wt%.
Kamonkhantikul et al. (2017) [62].	*n* = 112 specimens. 7 groups	• Zinc oxide ZnO. Size of 20–40 nm. • Silane coupling agent. • Heat-polymerized PMMA.	• One control group. • Three groups contain 1.25, 2.5, and 5 wt% ZnO, respectively. • Three groups contain 1.25, 2.5, and 5 wt% silanized ZnO, respectively.	• Flexural strength.	• The experimental groups’ flexural strength did not differ significantly. • Except for the 1.25% groups, the flexural strength of the silanized was significantly higher than that of the non-silanized with the same quantity of ZnO. • As the amount of ZnO increased, the flexural strength decreased.
Tijana et al. (2020) [63]	*N* = 24 specimens. 4 groups	• Gold Au. • Heat-polymerized PMMA.	• One control group. • Three groups contain 0.12, 0.43, and 0.74 wt% Au, respectively.	• Flexural strength. • Microhardness.	• The flexure strength in all experimental groups significantly decreased. • Microhardness values increased in all experimental groups. A significant increase showed only in 0.43 wt%.

**Table 2:** Mechanical properties of Glass Ionomer Cement reinforced by different NPs.

Author (Year)	Sample(s)	Type of NPs And Materials Used	Treatment Group(s)	Type of Tests	Outcomes
El-Negoly et al. (2014) [64].	*N* = 240 specimens. 10 groups.	• Titanium dioxide TiO_2_. Size of > 21 nm. • Conventional GIC. • Poly(acrylamide-co-sodium acrylate).	• One control group. • Three groups contain 3, 5, and 7 wt% copolymer, respectively. • Three groups contain 3, 5, and 7 wt% TiO_2_, respectively. • Three groups contain 3, 5, and 7 wt% of copolymer-TiO_2_, respectively.	• Compressive Strength. • Flexural strength. • Shear bond strength	• Compressive strength of groups that contain only copolymer decreased. • Incorporation of 3 wt% TiO_2_ significantly enhanced the compressive Strength as compared to the control group. Further increase in the content of TiO_2_ showed a reduction in the attained average compressive strength values. • Increasing the content of the copolymer to 5 wt% and 7 wt% significantly increased the mean values of the flexural Strength. • A significant increase in the flexural strength was observed upon incorporation of 3 wt% and 5 wt% TiO_2_, while further increase led to a significant reduction in the flexural strength. • Shear bond strength significantly increased upon incorporation of 3, 5, and 7 wt% copolymer, respectively. A very limited increase in the shear bond strength was observed upon addition of TiO_2_. • Using 7 wt% of copolymer-TiO_2_ led to a significant improvement in the compressive, flexure and shear bond strengths.
Garcia-Contreras (2015) [65]	*n* = 70 specimens. 9 groups.	• Titanium dioxide TiO_2_. Size of > 25 nm. • Three conventional types of GIC.	• One control group for each material. • Two groups contain 3 and 5 w/w TiO_2_, respectively for each material.	• Microhardness. • Flexural strength. • Compressive strength.	• A significant increase in microhardness, flexure and compressive strengths for the FX-II containing 3 and 5% TiO_2_ compared to control group. • Core shade cement improved only compressive strength significantly when 5% TiO_2_ were incorporated compared to control group. • Base cement did not show better properties significantly with the addition of TiO_2_.
Ibrahim et al. (2017) [66].	*N* = 172 specimens. 4 groups.	• Titanium dioxide TiO_2_. Size of ~ 21 nm. • Chitosan (CH) • Conventional GIC.	• One control group. • One group contains 3% w/w TiO_2_. • One group contains 10% v/v CH. • One group contains 3% w/w TiO_2_ and 10% v/v CH.	• Flexural strength. • Compressive strength. • Microhardness.	• For the flexural strength, TiO_2_-CH group showed significantly higher flexural strength compared to all the groups. In addition, CH group was statistically higher than the TiO_2_ group. • No significant difference was found in compressive strength between TiO_2_ group and TiO_2_-CH group. • No significant difference in the microhardness between control, TiO_2_ and TiO_2_-CH groups. However, the CH group showed significantly lower hardness compared to all other groups.
Hamid (2019) [67].	*n* = 12 specimens	• Titanium dioxide TiO_2_. Size of 10–20 nm. • Cetylpyridinium chloride monohydrate USP (CPC). • Conventional GIC.	• One control group. • One group contains 1% w/w CPC. • One group contains 3% w/w TiO_2_.	• Compressive strength.	• The compressive strength significantly increased with TiO_2_ group as compared with other groups. • No significant difference between CPC and control groups in the compressive strength.
Gjorgievska et al. (2015) [68].	*N* = 48 8 groups.	• Titanium dioxide TiO_2_. Size of 10–25 nm. • Aluminium oxide Al_2_O_3_. Size of > 100 nm. • Zirconium oxide ZrO_2_ Size of 80 nm. Two conventional types of GIC.	• One control group for each material. • Three groups contain 10% w/w TiO_2_, Al_2_O_3_ and ZrO_2_, respectively for each material.	• Compressive strength.	• In the case of GC Equia Fil material, using ZrO_2_ and TiO_2_ gave significantly higher compressive strengths compared to the control group. • In the case of Rock ChemFil material, only TiO_2_ gave significant higher compressive strengths compare to the control group.
Gjorgievska (2020) [69].	*N* = 96 specimens. 20 groups.	• Titanium dioxide TiO_2_. Size of 10–25 nm. • Aluminium oxide Al_2_O_3_. Size of > 100 nm. • Zirconium oxide ZrO_2_ Size of 80 nm. • Two conventional types of GIC.	• One control group for each material. • Three groups contain 2, 5 and 10% w/w TiO_2_, respectively for each material. • Three groups contain 2, 5 and 10% w/w Al_2_O_3_, respectively for each material. • Three groups contain 2, 5 and 10% w/w ZrO_2_, respectively for each material.	• Compressive strength.	• In the case of GC Equia Fil material, using 5 wt% Al_2_O_3_ and TiO_2_ gave lower compressive strengths relative to the control. While using 2 and 10 wt% Al_2_O_3_ show similar results with the control group but in case of ZrO_2_ and TiO_2_ show higher strength. The strength rising to a maximum with 10% loading. • In the case of Rock ChemFil material, using 5 wt% Al_2_O_3_ and ZrO_2_ gave lower compressive strengths relative to the control. While using 2 and 10 wt% Al_2_O_3_ and ZrO_2_ show similar results with the control group. while using TiO_2_ shows higher strength with all concentrations. • These values were not statistically significant.
Paiva et al. (2018) [41].	*n* = 32 4 groups.	• Silver Ag. • Conventional GIC.	• One control group. • Three groups contain 0.05, 0.1 and 0.5 wt% Ag, respectively.	• Compressive strength.	• All experimental groups show higher strength than control group. Using 0.5 wt% Ag gives significant increase in the compressive strength.
Jowkar et al. (2019) [70].	*N* = 120 3 groups.	• Silver Ag. Size of 20 nm. • Conventional GIC.	• One control group. • Two groups contain 0.1 and 0.2 wt% Ag, respectively.	• Microhardness. • Flexural strength. • Compressive strength. • Shear bond strength.	• Microhardness, compressive strength, and shear bond strength were significantly increased with increasing concentrations of Ag compared with control group. • The flexural strength of 0.2 wt% Ag was significantly increased compared with control group.
Chen et al. (2020) [71].	*n* = 78 6 groups.	• Graphene silver G-Ag. • Conventional GIC.	• One control group. • Five groups contain 0.05, 0.1, 0.5, 1 and 2 wt% G-Ag, respectively.	• Microhardness. • Flexural strength.	• A significant increase in the microhardness values in 0.05 and 0.1 wt% G-Ag groups compared with control group, while other groups show insignificant differences in the hardness. • A significant increase in the flexure strength in 0.1 wt% G-Ag group compared with control group. While other groups show insignificant differences in the flexure strength.
Barandehfard et al. (2016) [72].	*N* = 90 specimens 5 groups.	• Hydroxyapatite HA. Size of 20–30 nm. • Fluorapatite FA. Size of 20–30 nm. • Conventional GIC.	• One control group. • Four groups contain 5 and 8 wt% HA and FA, respectively.	• Diametral tensile strength. • Compressive strength. • Microhardness.	• All experimental groups showed higher compressive strength, diametral tensile strength and microhardness than control group. • Groups containing the FA in comparison with those containing HA display higher strengths.
Alatawi et al. (2019) [73].	6 groups	• Hydroxyapatite HA. Conventional GIC.	• One control group. • Five groups contain 1, 3, 5, 8 and 10 wt% HA, respectively.	• Compressive strength.	• Using HA with all concentrations gave higher strength than control group.
Sajjad et al. (2019) [74].	5 groups.	• Zirconia-Silica-Hydroxyapatite • ZrO_2_-SiO_2_-HA. • Conventional GIC.	• One control group. • Four groups contain 3, 5, 7 and 9 wt% ZrO_2_-SiO_2_-HA, respectively.	• Flexural strength. • Compressive strength.	• All ZrO_2_-SiO_2_-HA groups had higher flexural and compressive strength values than the control group. • 5 wt% ZrO_2_-SiO_2_-HA showed significantly higher flexural strength. • 5 and 7 wt% ZrO_2_-SiO_2_-HA showed significant higher compressive strength.
Sayyedan et al. (2014) [75].	5 groups.	• Forsterite Mg_2_SiO_4_. • Conventional GIC.	• One control group. • Four groups contain 1, 2, 3 and 4 wt% Mg_2_SiO_4_, respectively.	• Compressive strength. • Diametral tensile strength. • Flexural strength.	• Group of 3 wt% Mg_2_SiO_4_ showed highest compressive strength. Mg_2_SiO_4_ content less than 3 wt% does not have any significant effect on the compressive strength, while increasing the concentration decreases the compressive strength. • Group of 1 wt% Mg_2_SiO_4_ showed the highest flexural strength and diametral tensile strength.
Noori et al. (2019) [76].	*n* = 100 5 groups.	• Magnesium oxide MgO. • Conventional GIC.	• One control group. • Four groups contain 1, 2.5, 5 and 10 wt% MgO, respectively.	• Compressive strength. • Diametral tensile strength.	• A significant increase in the compressive strength and diametral tensile strength values only in 1 wt% MgO group compared with control group, while other groups show a decrease in the strengths.

**Table 3:** Mechanical properties of Resin composite reinforced by different NPs.

Author (Year)	Sample(s)	Type of NPs And Materials Used	Treatment Group(s)	Type of Tests	Outcomes
Xia et al. (2008) [77].	*N* = 25 specimens. 5 groups.	• Titanium dioxide TiO_2_. Size of > 20 nm. • Organosilane ATES. • Conventional composite resin.	• One control group. • Two groups contain 0.5 and 1 wt% un-silanized TiO_2_, respectively. • Two groups contain 0.5 and 1 wt% silanized TiO_2_, respectively.	• Microhardness. • Flexure strength.	• The microhardness of all experimental groups is increased by the addition TiO_2_. Groups of 1 wt% are significantly harder than those with only 0.5 wt%. silanized TiO_2_ groups are significantly harder than un-silanized groups. • The flexure strength of un-silanized TiO_2_ groups is less than control group. While silanized TiO_2_ groups show an increase in the strength than control group.
Al Jafary et al. (2019) [78].	*N* = 72 specimens. 4 groups.	• Titanium dioxide TiO_2_. Size of > 25 nm. • Flowable composite resin.	• One control group. • Three groups contain 1, 2 and 3 wt% TiO_2_, respectively.	• Microhardness. • Flexural strength.	• The microhardness and flexural strength of 1 wt% are significantly increased than control group. There is no significant difference in the strength of 2 wt% group. While the strength of 3 wt% is significantly decreased than control group.
Hojati et al. (2013) [79].	*N* = 60 specimens. 6 groups.	• Zinc oxide ZnO. Size of 20 nm. • Conventional composite resin.	• One control group. • Five groups contain 1, 2, 3, 4 and 5 wt% ZnO, respectively.	• Flexural strength. • Compressive strength. • Shear bond strength.	• No significant difference in the flexural strength of all experimental groups. • There is only a significant increase in the compressive strength and shear bond strength of 1 wt% group. while other groups have no significant differences.
Swetha (2019) [80].	*N* = 196 specimens. 7 groups.	• Zinc oxide ZnO. • Calcium fluoride CaF_2_. • Resin pit and fissure sealant.	• One control group. • Four groups contain 0.5 and 1 wt% ZnO and CaF_2_, respectively. • Two groups contain 0.5 and 1 wt% ZnO-CaF_2_, respectively.	• Compressive strength. • Flexural strength.	• The compressive strength of all experimental groups showed significantly higher than the control group. • The flexure strength of 0.5 wt% ZnO-CaF_2_ showed significantly higher than all other groups.
Balos et al. (2013) [81].	4 groups.	• Silicon dioxide SiO_2_. • Flowable composite resin.	• One control group. • Three groups contain 0.05, 0.2 and 1 wt% SiO_2_, respectively.	• Flexure strength. • Microhardness.	• The flexural strength of 0.05 and 1 wt% was higher than the control group. While adding 0.2 wt% gave lower flexure strength than control group. • The microhardness of 0.05 and 0.2 wt% was higher than the control group. While adding 1 wt% gave lower microhardness than control group. • Only 0.05 wt% group showed a significant increase in the flexure strength and the hardness.
Borat et al. (2020) [82].	*N* = 48 specimens. 8 groups.	• Farnesol loaded Halloysite Fa-HNT. • Flowable composite resin.	• One control group. • Seven groups contain 1, 3, 7, 10, 13, 17 and 20 wt% Fa-HNT, respectively.	• Flexural strength. • Compressive strength.	• The flexure strength and compressive strength values of 1,3 and 7 wt% groups showed higher than all other groups. • Using 7 wt% showed to be the optimum for enhancing the strengths.

**Table 4:** Mechanical properties of resin adhesive reinforced by different NPs.

Author (Year)	Sample(s)	Type of NPs And Materials Used	Treatment Group(s)	Type of Tests	Outcomes
Gutierrez et al. (2017) [83].	*N* = 35 specimens. 7 groups.	• Copper Cu. Size of 40–60 nm. • Ambar etch and rinse adhesive system.	• One control group. • Six groups contain 0.0075, 0.015, 0.06, 0.1, 0.5 and 1 wt% Cu, respectively.	• Tensile strength. • Microtensile bond strength.	• No significant differences in tensile strength between groups. • A significant increase in the microtensile bond strength was observed in the 0.1 and 0.5 wt% groups. • A significant decrease in the microtensile bond strength after 1 year of water storage was only observed for the control group. While the experimental groups showed similar tensile bond strength between the immediate and 1-year periods.
Gutierrez et al. (2019) [84].	*N* = 60 specimens. 6 groups.	• Copper Cu. Size of 40–60 nm. • Zinc oxide ZnO. Size of 10–30 nm. • Prime & Bond Active universal adhesive system. • Ambar universal adhesive system.	• One control group for each material. • One group contains 5 wt% ZnO and 0.1 wt% Cu for each material. • One group contains 5 wt% ZnO and 0.2 wt% Cu for each material.	• Microhardness. • Microtensile bond strength.	• In the case of Prime & Bond Active, no significant differences between all groups were detected. • In the case of Ambar universal adhesive, a significant increase in the microhardness of all experimental groups compared with control group. • For both adhesive materials, no significant differences were observed in the microtensile bond strength among all groups.
Torres-Rosas (2020) [85].	*N* = 31 specimens. 2 groups.	• Copper Cu. Size of 2–10 nm. • Adper etch and rinse adhesive system.	• One control group. • One group contains 0.01 wt% Cu.	• Shear bond strength.	• A significant increase in the shear bond strength of experimental group than control group.
Sadat-Shojai et al. (2010) [86].	*N* = 98 specimens. 7 groups.	• Hydroxyapatite HA. • “Ethanol, UDMA, Bis-GMA, HEMA, TMPTMA” as experimental adhesive. • Adper etch and rinse adhesive system.	• One control group contains Adper. • One group contains adhesive without HA. • Five groups contain 0.2, 0.5, 1, 2 and 5 wt% HA, respectively.	• Diametral tensile strength • Flexure strength. • Shear bond strength.	• A significant increase in the diametral tensile strength and flexural strength of 0.2 and 0.5 wt% HA. • A significant decrease in the flexural strength of 5 wt% HA. • A significant increase in the shear bond strength of 0.2 wt% HA.
Zhang et al. (2013) [87].	*n* = 12 specimens. 4 groups.	• Silver Ag. • MDPB. • SBMP primer.	• One control group. • One group contains 5 wt% MDPB. • One group contains 0.05 wt% Ag. • One group contains 5 wt% MDPB and 0.05 wt% Ag.	• Shear bond strength.	• No significant differences in the shear bond strength between all groups.

**Table 5:** Mechanical properties of orthodontic adhesive reinforced by different NPs.

Author (Year)	Sample(s)	Type of NPs And Materials Used	Treatment Group(s)	Type of Tests	Outcomes
Argueta-Figueroa et al. (2015) [88].	*N* = 60 specimens. 2 groups.	• Copper Cu. • Conventional orthodontic adhesive.	• One control group. • One group contains 0.01 wt% Cu.	• Shear bond strength.	• A significant increase in the shear bond strength of 0.01 wt% Cu.
Felemban et al. (2017) [89].	*N* = 60 specimens. 3 groups.	• Zirconium dioxide ZrO_2_. Size of 70–80 nm. • Titanium dioxide TiO_2_. Size of > 50 nm. • Conventional orthodontic adhesive.	• One control group. • Two groups contain 0.5 and 1 wt% ZrO_2_-TiO_2_.	• Compressive strength. • Diametral tensile strength. • Shear bond strength.	• A significant increase in the compressive strength, tensile strength, and shear bond strength of ZrO_2_-TiO_2_ groups than control group.

**Table 6:** Mechanical properties of endodontic sealer reinforced by different NPs.

Author (Year)	Sample(s)	Type of NPs And Materials Used	Treatment Group(s)	Type of Tests	Outcomes
Viapiana et al. (2014) [90].	*N* = 30 specimens. 5 groups.	• Zirconium dioxide ZrO_2_. • Niobium oxide NbO. • Portland Cement. • AH Plus. • Sealapex. • MTA Fillapex.	• Three groups contain AH Plus, Sealapex and MTA Fillapex, respectively. • One group contains Portland cement with 30 wt% ZrO_2_. • One group contains Portland cement with 30 wt% NbO.	• Compressive strength.	• At 24 h, AH Plus, Portland cement with ZrO_2_ and NbO groups have the highest compressive strength. • At 21 days, AH Plus has significant highest strength, followed by Portland cement with ZrO_2_ and NbO. • MTA Fillapex had the lowest compressive strength at both time intervals. • Sealapex was not subjected to testing, because the group failed to set.
Barros et al. (2014) [91].	*n* = 30 specimens. 6 groups.	• Quaternary ammonium polyethylenimine NPs QPEI. Size of 58 ± 18 nm. • AH Plus. • Pulp Canal Sealer EWT.	• One control group for each material. • Two groups contain 1 and 2 wt% QPEI, respectively for each material.	• Compressive strength.	• No significant differences in the compressive strength of all experimental groups.

**Fig. 2:**
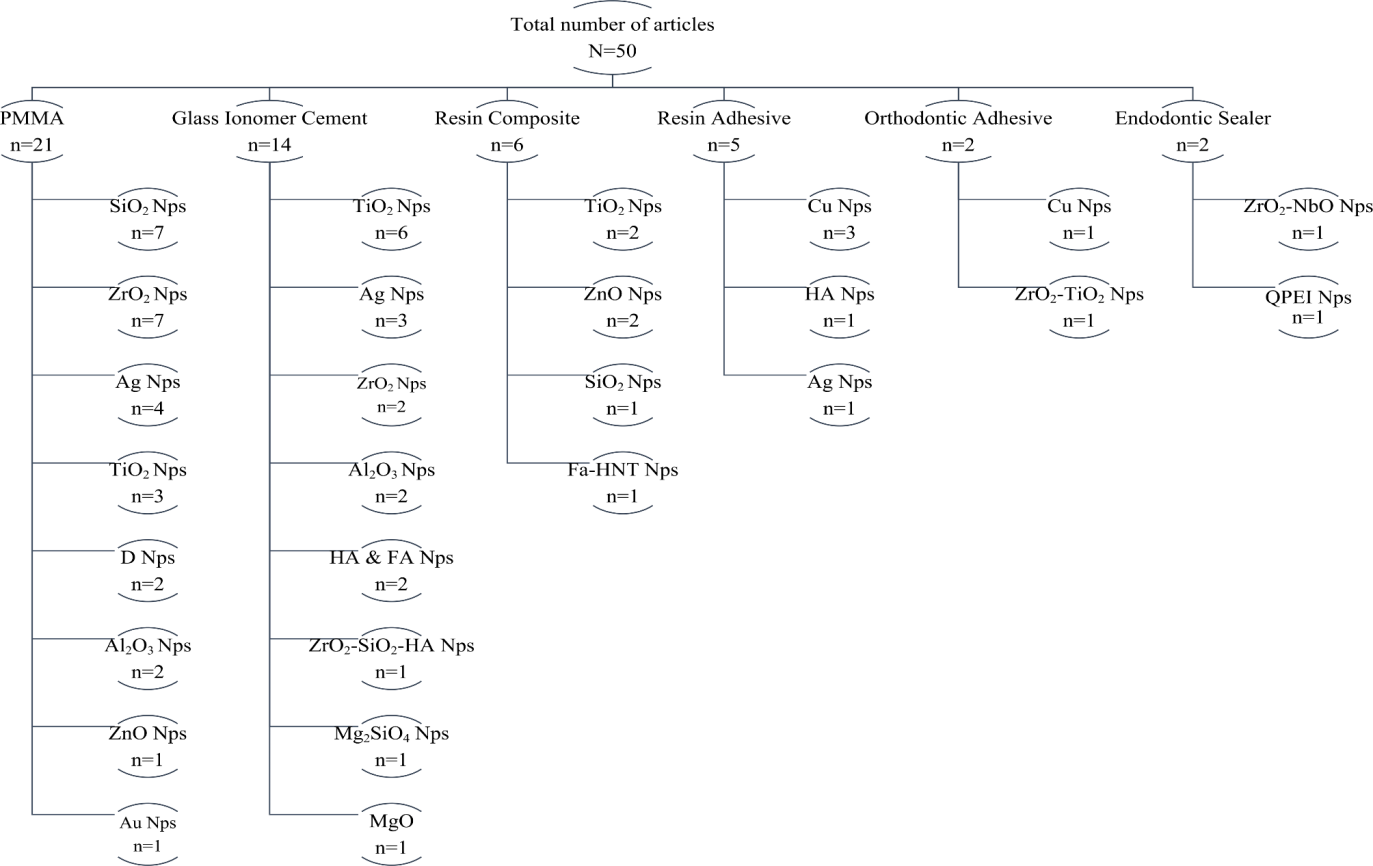
Diagram of the distribution of articles by the dental material and type of nanoparticle

The total number of articles included in this review is 50 articles as shown in Fig. 2. The most common material that was investigated by researchers was PMMA with a total of 21 studies, followed by glass ionomer cement (GIC), resin composite, resin adhesive, and orthodontic adhesive with a total of 14, 6, 5 and 2 studies, respectively. The least investigated material was endodontic sealer with two articles. The most commonly used NPs used in all the studies were SiO_2_, TiO_2,_ and ZrO_2_.

## DISCUSSION

All 50 reviewed studies were in-vitro bench studies that used inorganic NPs as enforcing fillers in different dental materials to improve their mechanical properties. Their main objective was focused on enhancing the mechanical properties of some of the widely used conventional dental materials as those materials possessed some limitations. In order to improve those mechanical properties, the researchers resorted to using inorganic NPs as fillers. The studies then assessed the different mechanical properties of those NPs-incorporated dental materials with different concentrations of the inorganic NPs in an attempt to formulate a hybrid material with superior mechanical properties to the conventional one. However, the most challenging factor that these studies faced when incorporating the NPs with the dental materials is the tendency of NPs to agglomerate. This is a serious problem that can lead to a reduction of the nanoparticles’ surface energy, resulting in a loss and change of their nano-properties [7]. For that reason, and to achieve an appropriate homogeneous dispersion, all researchers used the NPs only with low levels of concentrations as it aided to minimize or eliminate the NPs agglomeration. In addition, some researchers found that using a silane coupling agent with the NPs was highly effective in avoiding that issue [45, 54, 56, 62, 77, 92]. The outcome of the mechanical properties of the modified dental materials reviewed in this study varied from one material to another, and from one mechanical testing to the other, based on the type and concentration of the NPs used, together with the original inherit composition and characteristics of the materials themselves.

### Polymethyl methacrylate (PMMA)

PMMA-based resins are widely utilized in dentistry for a variety of applications, including removable base plates, functional appliances, and denture bases [93]. Its benefits stem from its biocompatibility and esthetics [81, 94]. It is also simple to manufacture, inexpensive, and has specific characteristics such as low weight, low water sorption, and low solubility [45, 95]. The material’s weakness involves the limited mechanical strength of PMMA resin denture base materials, with low impact and flexural strengths [94, 96, 97]. As a result, denture failure occurs frequently when eating or falling [98]. For these reasons, PMMA was the top material on the list of most studies (21 studies) that used NPs as a reinforcing filler to strengthen the PMMA’s mechanical properties. The reinforcing agent’s chemical bond with the polymer matrix was strong enough to withstand and transmit occlusal forces from the weaker polymer to the stronger reinforcing agent [51, 52, 55, 56, 59–61]. Additionally, the consistent impregnation of the reinforcing agent in the matrix inhibits the formation of stress concentrators [50, 51, 55, 56, 61] which could compromise the resins’ mechanical qualities.

The most commonly used NPs as fillers to reinforce PMMA were SiO_2_ and ZrO_2_ (7 studies each). The purpose for selection of those two NPs was due to their inherent properties that can enhance acrylic resins’ mechanical characteristics [50, 52, 54]. They possess a number of desirable features, including high toughness, mechanical strength, abrasion and corrosion resistance, and biocompatibility [99, 100]. Additionally, ZrO_2_ has excellent mechanical qualities that allow it to resist crack propagation, and it is noted to hold the greatest hardness of any oxide [101, 102]. The flexure strength, tensile strength, impact strength, fracture toughness and the surface hardness of PMMA reinforced by 0.5 to 1 wt% of ZrO_2_ or SiO_2_ increased significantly [45, 46, 49, 50, 52]. Surface treated ZrO_2_ or SiO_2_ with a silane coupling agent led to a decrease in the surface tension of the particles and influenced the spatial distribution of fillers, resulting in higher mechanical properties than with untreated NPs [51, 56, 92].

Conversely, one study concluded that using SiO_2_ incorporated with PMMA led to a significant decrease in the flexure strength of the original material. However, while further examining their technique, it was found that they incorporated the NPs in the monomer liquid and that they mixed it manually. That approach can cause uneven distribution and dispersion of the NPs within the matrix, which was confirmed by their SEM analysis that detected porosity in the PMMA matrix [47].

Although there is less published work on using Al_2_O_3_, TiO_2_ and diamond NPs fillers with PMMA compared to ZrO_2_ and SiO_2_, the results of those studies showed similar significant increase in the flexure strength, impact strength, and surface hardness [48–50, 60, 61]. However, due to the variability of the NPs, more studies with those NPs are needed to verify and validate those results.

Using Ag NPs did not appear to improve the mechanical properties of PMMA [57, 58, 103]. Ag NPs is mainly used for their antimicrobial activity to treat common infections of oral mucosal tissues in complete denture users [104]. They have been demonstrated to be effective against many microorganisms such as *E. coli, Staphylococcus aureus, Staphylococcus epidermidis, Candida albicans, and Streptococcus mutans* [105–107]. However, a significant increase in the flexure strength of PMMA was found when it was modified with graphene-Ag NPs [59] Additionally, adding Au NPs to PMMA showed no significant decrease in the flexure strength of the material; however, a significant increase in the micro hardness of the PMMA was observed when 0.43 wt% Au was added [63]. Similar results regarding the flexure strength was observed when ZnO was incorporated with PMMA [62].

The overall results of those studies indicated that most NPs proved to have a positive significant effect in improving the different mechanical properties of PMMA, especially when used in low concentrations of 0.5%.

### Glass ionomer cement (GIC)

Biological compatibility, adhesion to moist tooth structure that allows for little removal of sound tooth structure, and anticariogenic qualities due to fluoride release are just a few of the benefits of GIC. Furthermore, it has a coefficient of thermal expansion that is similar to that of tooth structure. Despite those advantages, conventional glass ionomer cements possess limitations as restorative materials due to their brittleness, low flexural strength, low fracture toughness, low wear resistance, slow setting rate, high solubility and the relatively high sensitivity to water at the initial stage of setting [106, 107]. Numerous modifications had been developed over the years in attempt to overcome these drawbacks and improve the mechanical properties [108]. Most recently, NPs have been incorporated into glass ionomers with the objective of enhancing their mechanical strength. GIC was the second in list of number of studies (14 studies) that used NPs to augment its properties.

TiO_2_ was the top NPs selection used for GIC. Because of the relatively smaller size of TiO_2_ NPs supplemented into the glass powders, they can fill in the voids between the bigger GIC glass particles and serve as extra polyacrylic polymer bonding sites [35, 64–69]. For this reason, the flexure strength, compressive strength, and hardness value increased significantly by incorporation of 3, 5 and 7 wt% TiO_2_ [64–69]. Moreover, the compressive strength of GIC reinforced with 2 and 10 wt% ZrO_2_ also increased, while the addition of Al_2_O_3_ showed no effect on the mechanical properties [68, 69].

Together with the exceptional antimicrobial properties of Ag NPs, the incorporation of Ag to GIC showed a significant increase in the hardness, flexure strength, compressive strength, and shear bond strength [70–72]. The concentrations of 0.1–0.5 wt% were the most optimum concentrations for increasing those mentioned properties. At that very low level of concentration, the voids in the GIC matrix were filled with the small size of Ag nanoparticle fillers [35, 68, 70–72, 109]. Filling those voids resulted in the improved packing of particles within the matrix, which ultimately led to the enhancement of those mechanical properties.

Enforcing GIC with hydroxyapatite (HA) and fluorapatite (FA) NPs were evaluated in other studies [73, 74]. HA has a comparable composition and structure to enamel and dentin [109–111], which gives it the advantage and the edge to enhance the shear-bond strength with tooth structure. Furthermore, compressive strength, diametral tensile strength, and microhardness had all been significantly improved [73, 74] Alternatively, FA showed better results than HA due to its higher crystallinity [111, 112].

A combination of HA and SiO_2_ have been successfully used to enhance GICs [113]. Moreover, ZrO_2_ or a combination of HA and ZrO_2_ had been incorporated in attempts to strengthen GIC with improved outcomes [114, 115]. Zirconium and its oxide, due to their good dimensional stability and toughness, have been widely used for the toughening and strengthening of brittle HA bio-glasses in biomedical applications [116]. Because of that, using 5 or 7 wt% of ZrO_2_-SiO_2_-HA with GIC resulted in a significant improvement in the mechanical properties.

The crystalline structure of forsterite NPs resulted in the production of crystalline phases in the amorphous cement matrix [109, 117]. This could justify the enhancement of the flexure strength, compressive strength, and tensile strength of GIC reinforced with 1 wt% Mg_2_SiO_4_ [75].

Incorporation of MgO nanoparticles into different dental products was done to imbue antimicrobial properties [76, 118–120]. Regarding its mechanical effect on the GIC, the results showed a significant increase in the compressive strength and diametral tensile strength values when MgO was used at 1 wt% [77].

In general, GIC’s limited mechanical properties were improved when modified with different NPs in all the in-vitro studies reviewed. A low concentration of 0.1-1% of different NPs was found to give the most optimum results. Furthermore, comparable composition and structure of NPs to those of enamel and dentin significantly improved the GIC’s different mechanical properties, particularly the ones with higher crystallinity.

### Resin composite

Resin-based composites are currently one of the most popular dental restorative materials due to their superior esthetic features and good adhesive properties [121–123]. However, resin composites have a number of mechanical shortcomings, including wear resistance, hardness, and shrinkage tendency [121, 124]. Secondary caries continues to be the most common cause of dental restorations’ service life being shortened, ultimately necessitating restorative material replacement. In terms of improving the features of resin composites, there are a few solutions that could be achieved. Dental resin nanocomposites are one of them; they are made up of a resin matrix, nanofillers, photo-initiator, and other components that are integrated together.

Regarding the benefits and drawbacks of nanocomposites, manufacturers rarely disclose the accurate proportional quantity, geometry, and size of nanofiller, which can be troublesome when these are important factors in determining the outcome of the mechanical properties. Furthermore, mostly all restorative “nanocomposites” are “nano-hybrids” with substantially larger volume ratios of non-nano sub-micron or micron-sized particles [7]. The other way in order to improve the mechanical properties of conventional resin composite is to use NPs [77–83]. NPs with nano-scale dimensions allow a wider area of interactions with microorganisms, thereby increasing their antibacterial activities, which is a requisite for dental restorative materials to prevent secondary caries [79, 80, 125]. Most of the studies involving the addition of NPs to dental composite resins mainly focused on their anti-bacterial effects, while the information regarding their mechanical properties was limited.

The mechanical properties, radiopacity, and optical properties of conventional composite resins have been improved by addition of inorganic NPs such as ZnO and TiO_2_ [77–81]. TiO_2_ offers a wide range of positive features. It is nontoxic, chemically stable, and has high photocatalytic efficiency [77]. In addition, TiO_2_ NPs are tooth colored and does not stain the restoration [78, 126]. The presumably smaller size of theTiO_2_ NPs promotes close cross-linking to the resin particles and prevents their degradation [79, 127]. Using 1 wt% TiO_2_ with dental resin composite leads to a significant increase in the flexure strength and surface hardness [78, 79]. These results increased specifically when the NPs were treated with a silane coupling agent, improving the dispersal and bonding of the filler particles throughout the matrix [78]. Additionally, the supplementation of Fa-HNT based fillers at low concentrations in dental composites was found to greatly improve the mechanical properties [82].

ZnO showed no effect on the flexure strength, but had a significant effect on the compressive strength and flexure modulus at 1 wt% concentration [80]. Combining ZnO with CaF_2_ showed a significant increase in the flexure strength at 0.5 wt% [81]. The opacity of ZnO NPs against visible light may have had a negative impact on light curing and, as a result, the mechanical characteristics of composites [80, 81, 86]. Because of that, further increase in the concentration of NPs led to decrease in the mechanical properties. It is possible to deduce that the decrease in mechanical characteristics was more likely a result of the effect of the NPs on composite curing rather than the occurrence of structural defects owing to particle agglomeration.

Despite the few number of studies that assessed the effect of enhancing composite resin with NPs (6 studies), their outcome was in favor of the positive added value of NPs in improving the tested mechanical properties of resin composite.

### Resin adhesive

Dental adhesives have become commonly employed in restorative dentistry due to their esthetic and conservative characteristics. In reality, the clinical efficacy of resin composite is influenced by the restorative materials’ full adherence to enamel and dentin [95]. The acid-etch process is frequently successful when it comes to enamel [128, 129]. Dentin, on the other hand, is a hydrated biological composite made up of inorganic compounds, organic compounds, and water, with capabilities that vary greatly depending on where it is found. Furthermore, dentin has fluid-filled dentinal tubules, which create a dynamic and wet surface for bonding chemicals, as well as a more difficult situation than enamel [130, 131]. NPs have recently been introduced into dental adhesives with the goal of increasing mechanical qualities [132, 133]. Filler particles are used in dental adhesives to strengthen the bond between the adhesive and the dentin by entering the tubules of the dentin, reducing polymerization shrinkage, and raising the elastic modulus of the adhesive layer. [134]

Adding HA NPs to dental adhesives showed an increase in the micro-shear bond strength, tensile and flexure strengths [86]. Moreover, using Cu NPs demonstrated a better shear bond strength than conventional resin [83–85]. When compared to Cu free adhesives, these adhesives formed interfaces capable of reducing the deterioration of resin–dentin bonded surfaces [134]. Furthermore, since the collagen crosslinking enzyme Lysyl oxidase (LOX) is Cu dependent, Cu NPs had an indirect effect as a crosslinking agent, which consequently increased the strength of the collagen network, one of the components of the hybrid layer. Copper’s activity as a cross-linker may help collagen become more resistant [135, 136]. Using Ag NPs did not give any significant results on the mechanical properties of dental resin adhesive [87].

### Orthodontic adhesive resin

Failure of the orthodontic bracket bonding method results in frequent debonding of the brackets, delaying treatment results. The bonding mechanisms and the failure rates of orthodontic brackets might be affected by tooth or material-related variables [136]. Previous studies have concentrated on the pre-treatment of resin monomers [137], inorganic fillers, and the development of curing procedures to improve the properties of orthodontic resin adhesives [110, 125, 138–140]. In dental adhesives, NPs have been explored as strengthening fillers—adding these NPs will result in an increase of the adhesive’s mechanical properties [88, 89, 137, 141]. Using Cu NPs with the orthodontic adhesive resulted in a significant increase in the shear bond strength [88]. Mixing of ZrO_2_ with TiO_2_ showed a significant increase in the shear bond strength, compressive strength, and tensile strength of the orthodontic adhesive [89].

### Endodontic sealer

The physicochemical and biological features of the newly proposed root canal filler materials should be investigated. Setting time, flow, film thickness, solubility, radiopacity, dimensional stability, and compressive strength of endodontic sealers are among the criteria evaluated by the American National Institute/American Dental Association and the International Organization for Standardization [142, 143]. Combining ZrO_2_ with NbO NPs showed a significant increase in the compressive strength in Portland sealer compared to MTA Fillapex and Sealapex. However, the compressive strength of AH Plus sealer was significantly higher than reinforced Portland cement [90]. Using QPEI NPs with AH Plus and Pulp Canal Sealer resulted in no significant difference in the mechanical properties between all experimental groups [91]. Nonetheless, due to lack of sufficient studies on measuring the mechanical properties of sealer materials reinforced with NPs, we cannot give a conclusive statement about using NPs in different endodontic sealers.

### Analysis of NPs used

The incorporation of NPs into different dental materials was utilized as a positive means of increasing their mechanical properties. TiO_2_ NPs offered flexure strength, impact strength, and surface hardness when incorporated into PMMA, higher flexure and compressive strengths with GIC, and enhanced the flexure strength and the surface hardness of the resin composite. ZrO_2_ and SiO_2_ offered better flexure strength, tensile strength, impact strength and surface hardness properties when incorporated into PMMA and enhanced the flexure strength and compressive strength of GIC. The shear bond strength of Cu nanoparticle-modified dental adhesive material was significantly higher when compared with the original material. The improvement in the mechanical properties of different dental materials that included NPs occurred due to several reasons: these NPs filled the empty voids within the matrix of the original materials, which improved their strength. Furthermore, the large interfacial area of NPs provided more contact points with the materials, and also interrupted crack propagation by transferring stresses from the weak original material to the strong NPs filler. Additionally, using silane-coupling agent created a strong adhesion between the original material matrix and the NPs. Regarding resin composite materials, the small size of NPs promoted cross-linking to the resin particles. Using NPs with adhesive resin improved the mechanical properties due to an increase in the strength of the collagen network.

All of the reviewed studies dealing with NPs-reinforced dental materials were in-vitro studies. Many of the studies operated on the basis that the use of various inorganic metal oxide NPs has the potential to be an innovative solution to improve those materials’ weaknesses. Despite the great potential that their promising results revealed for the effectiveness of incorporating NPs in different dental materials, unfortunately those bench studies were not followed up yet with clinical trials that could support those findings. Currently, there are very few studies that performed in-vivo testing in that context. Hence, those in-vitro results cannot be generalized to the real clinical settings without the confirmation of many and different in-vivo studies. NPs can be a great contemporary addition that could provide those dental materials tested with superior properties compared to those of the original ones. They can provide those enhanced materials with longevity and a higher success rate, and consequently augment their quality along with enhancing the quality of life of the patients using them. Therefore, well-designed clinical trials are essential to confirm the results of the published in-vitro claims and to encourage manufacturers to include NPs as a standard ingredient in the composition of those materials.

## Summary

Several of the in-vitro studies demonstrated that the incorporation of NPs within various dental materials revealed very promising significant results in improving the different mechanical properties of the original material. Nonetheless, due to the variety of the nanoparticles, it is challenging to identify the optimum specifications that suit the spectrum of dental materials. Generally, the studies used NPs in low concentrations less than 1% by weight along with a silane coupling agent to minimize agglomeration issues; however, further clinical trials can validate the claimed positive results and confirm the performance and long-term effectiveness of those new hybrid nano materials in real clinical settings.

## Data Availability

Data is available from the corresponding author upon reasonable request.

## Code Availability

Code sharing is not applicable to this article as no code was generated or analyzed during the current study.

## Abbreviations

NPs: Nanoparticles
PMMA: Polymethyl methacrylate
HA: Hydroxyapatite
FA: Fluorapatite
GIC: Glass ionomer cement
QPEI: Quaternary ammonium polyethyleneimine
